# The Dopaminergic Midbrain Encodes the Expected Certainty about Desired Outcomes

**DOI:** 10.1093/cercor/bhu159

**Published:** 2014-07-23

**Authors:** Philipp Schwartenbeck, Thomas H. B. FitzGerald, Christoph Mathys, Ray Dolan, Karl Friston

**Affiliations:** 1 The Wellcome Trust Centre for Neuroimaging, UCL, LondonWC1N 3BG, UK; 2 Centre for Neurocognitive Research and Department of Psychology, University of Salzburg, Salzburg, Austria; 3 Neuroscience Institute and Centre for Neurocognitive Research, Christian-Doppler-Klinik, Paracelsus Medical University Salzburg, Salzburg, Austria; 4 Max Planck UCL Centre for Computational Psychiatry and Ageing Research, London, UK

**Keywords:** active inference, confidence, dopamine, neuroeconomics, precision

## Abstract

Dopamine plays a key role in learning; however, its exact function in decision making and choice remains unclear. Recently, we proposed a generic model based on active (Bayesian) inference wherein dopamine encodes the precision of beliefs about optimal policies. Put simply, dopamine discharges reflect the confidence that a chosen policy will lead to desired outcomes. We designed a novel task to test this hypothesis, where subjects played a “limited offer” game in a functional magnetic resonance imaging experiment. Subjects had to decide how long to wait for a high offer before accepting a low offer, with the risk of losing everything if they waited too long. Bayesian model comparison showed that behavior strongly supported active inference, based on surprise minimization, over classical utility maximization schemes. Furthermore, midbrain activity, encompassing dopamine projection neurons, was accurately predicted by trial-by-trial variations in model-based estimates of precision. Our findings demonstrate that human subjects infer both optimal policies and the precision of those inferences, and thus support the notion that humans perform hierarchical probabilistic Bayesian inference. In other words, subjects have to infer both what they should do as well as how confident they are in their choices, where confidence may be encoded by dopaminergic firing.

## Introduction

Dopamine plays a key role in learning and decision making. It has been linked to signaling of expected utility (Fiorillo et al. 2003), salience (Berridge 2007), stimulus-novelty (Bunzeck and Düzel 2006; Redgrave and Gurney 2006) and reward prediction error (RPE; i.e., the mismatch between the predicted and actual reward) (Schultz et al. 1997; Steinberg et al. 2013). However, a more general account of dopaminergic activity, which provides a principled explanation for all these associations, has yet to be established.

Recently, we formulated an inference scheme that attempts a unified explanation for the role of dopamine in choice (Friston et al. 2013). Here, planning and decision making are understood as approximate Bayesian (active) inference (Toussaint and Storkey 2006; Botvinick and Toussaint 2012), where agents minimize their surprise about future states. In this scheme, valuable policies (i.e., sequences of control states) minimize the relative entropy (Kullback–Leibler [KL] divergence) between the probability distributions over likely and desired outcomes. The value of a policy reflects how close the distribution of likely outcomes is to the desired distribution (see Friston et al. (2013) for a detailed discussion—for related ideas about decision making and KL control, see Kappen et al. (2009); Todorov (2009)).

A key consequence of this perspective is that, to minimize surprise, agents need to estimate the precision of (confidence in) their inferences or beliefs about the relative values of policies. It is therefore not sufficient to represent the desired outcome and how to get there. Instead, one also has to optimize the expected certainty or precision that the desired outcome can be reached. Expected precision needs to be widely broadcast, since it plays a crucial role in hierarchical inference (Friston et al. 2010; Friston 2011). Locally, precision plays a modulatory (multiplicative) role to select or gate message passing in Bayesian belief updating schemes. These features are anatomically and neurophysiologically consistent with the encoding of precision by neuromodulators (Friston et al. 2012; Moran et al. 2013) and with dopaminergic activity in particular, based on its key role in decision making and its unique functional anatomy (Friston et al. forthcoming). Precision is now emerging as an important interpretation of empirical brain responses as measured with fMRI (see Iglesias et al. 2013). Furthermore, when approximate inference is performed using Variational Bayes (Attias 2000; Beal 2003), the time course of precision updating closely resembles phasic dopamine responses (Friston et al. forthcoming). This perspective on the functional role of dopamine fits comfortably with formulations in terms of signal to noise (Cohen and Servan-Schreiber 1992; Goldman-Rakic 1998), uncertainty and precision (Fiorillo et al. 2003; Fiorillo et al. 2008), and the key role that dopamine plays in selecting among alternative actions (Braver et al. 1999; Frank 2005; Humphries et al. 2009; Cisek and Kalaska 2010). This is because precision increases with signal to noise and decreases with uncertainty. In particular, the precision of beliefs about completing actions manifests in the efficiency of action selection, often conceived of in terms of winner take all mechanisms.

To test the hypothesis that dopamine encodes precision, we designed an event-related fMRI experiment, in which subjects were given the option of accepting a current offer or waiting for a potential higher offer in the future, but with the risk of losing everything. In this setting, precision can be understood as the confidence that a high offer will be accepted in the future—and can be easily manipulated in this setup. Although we cannot measure dopaminergic activity directly in human subjects, we used activity in midbrain regions that include the ventral tegmental area (VTA) and substantia nigra (SN) (origin of dopaminergic projections) as a proxy. To optimize choice behavior in this particular task, precision itself has to be optimized to ensure optimal selection of the most valuable policy. In short, our task allowed us to test whether activity in SN/VTA reflects expected precision.

The active inference scheme evaluated in this work follows from the principle of minimizing variational free energy (Friston et al. 2006). It can be thought of as the discrete time version of predictive coding—that now predominates in models of perceptual inference and learning (Srinivasan 1982; Rao and Ballard 1999; Seth 2011; Bastos et al. 2012). Specifically, it uses variational Bayes to solve discrete time and Markov decision problems, in the same way that predictive coding uses Bayesian filtering with continuous time and states. To model purposeful behavior, we simply assume that agents are equipped with prior beliefs that they will minimize the difference between predicted and desired outcomes; either using (discrete) variational message passing (Friston et al. 2013) or (continuous) predictive coding (Friston, Adams et al. 2012).

It is important to note that our task requires planning or inference, not reward learning. In other words, subjects knew the contingencies in advance—and there was no learning or updating of value functions that would call upon RPE (Schultz et al. 1997). Our hope was to establish a role of dopamine in the context of inference—searching for evidence that dopamine encodes the precision or confidence during online assimilation (i.e., the assimilation of sensory data to optimize estimates of hidden states generating outcomes) and decision making under a biologically plausible model (Friston et al. 2013, forthcoming). This is potentially important because the physiology of dopaminergic neurotransmission is more consistent with a role in mediating precision. Furthermore, changes in (inverse) precision are identical to (negative) changes in expected value. This means that changes in precision may provide a sufficient account of the reward-dependent aspects of dopaminergic responses.

In summary, the problem faced by our subjects was to infer what they were most likely to do, if they were to end up in desirable states—and then act on that inference. On the basis of previous (theoretical) work, we anticipated that midbrain dopaminergic areas and their projection fields would correlate with trial-by-trial changes in the precision or confidence in those inferences.

## Materials and Methods

### Participants

Twenty-six right-handed participants (15 females) with normal or corrected–to-normal vision and no known history of neurological or psychiatric disorders participated in our study. All participants were university students with a mean age of 28 (standard deviation = 8.69, range = [20, 55]) and were recruited via the University College London Psychology Subject Pool. Subjects were told that the minimum payment for participating would be £25 but payment could be increased according to their performance—the average “win” was £37. Two participants were excluded from the analysis due to misses in more than 10% of games. The study was approved by the UCL Research Ethics Committee for fMRI-studies with performance-dependent payment (Approval-Code: 3450 002) and written informed consent was secured from all participants.

### Behavioral Paradigm

Subjects underwent a 1-h training phase followed by one and a half hours of scanning. Subjects performed 3 sessions comprising 36 games outside the scanner and the same number of sessions and games inside the scanner—where each session lasted for about 18 min. The objective of the extensive prescan training was to over-train subjects on the contingencies of the task and therefore minimize changes in behavior due to learning. This ensured the task could be treated as a pure inference problem.

Subjects had to solve a limited offer task, which required a choice between accepting an offer currently available or waiting for a higher offer in the future—with the risk of losing everything. Each game comprised successive trials (time steps) and subjects had to make a decision at each trial. At the first trial of a game, subjects were offered a specific monetary amount (in pence) and had to decide whether to accept this offer or wait for the next trial. If an offer was accepted, subjects won the accepted amount and had to wait for the remaining trials of that game, without being able to choose again (this ensured that subjects did not accept prematurely to increase the number of games). If subjects decided to wait, then the initial offer could be retained, it could be withdrawn or it could be replaced by a high offer. If the initial offer remained, subjects had to make the same choice again. If the offer was withdrawn, then the monetary amount on the screen was replaced by a cross, indicating that subjects had won nothing and would have to wait for the remaining trials to play out. If the initial offer was replaced by a high offer, subjects accepted immediately.

The probabilities for a withdrawal (*r*) and for receiving the high offer (*q*) were defined by hazard rates, such that for the 2 actions (accept or wait), the transition probabilities over states (*s*) and actions (*a*) were as follow:

$$P(s_{t+1}|s_{t},a_{\mathrm{accept}})=\left[\begin{array}{ccccc} 0 & 0 & 0 & 0 & 0\\ 0 & 0 & 0 & 0 & 0\\ 0 & 0 & 1 & 1 & 1\\ 1 & 0 & 0 & 0 & 0\\ 0 & 1 & 0 & 0 & 0 \end{array}\right]$$

$$P(s_{t+1}|s_{t},a_{\mathrm{wait}})=\left[\begin{array}{ccccc} P(\text{initial}) & 0 & 0 & 0 & 0\\ P(\text{high}) & 0 & 0 & 0 & 0\\ P(\text{withdrawal}) & 1 & 1 & 0 & 0\\ 0 & 0 & 0 & 1 & 0\\ 0 & 0 & 0 & 0 & 1 \end{array}\right]$$

with

P(initial)=1−(r+q)

$$P(\text{high})=q=(1-r)*(1-(1-0.5){)}^{1/T}$$

$$P(\text{withdrawal})=r=1-{\left(1-\frac{1}{22}\right)}^{t}$$

where *t* refers to the current trial and *T* is the number of trials in a game. This implies that a high offer is less likely as the game proceeds and one is more likely to lose the initial (lower) offer.

We varied the initial offer and the length of a game (number of trials). The amount of the initial offer was drawn from either a low or a high uniform distribution—with a mean of 12 (range: 9–15) and 32 (range: 29–35) pence, respectively. In every game, this initial offer could be replaced by a high offer, which was always 80 pence. Furthermore, we compared long and short games, where long games consisted of 8 trials and short games of 4 trials. Each session was equally divided into long and short games with a low or high initial offer, respectively—providing a total of 9 games per session for each of these 4 conditions.

**Figure 1.**
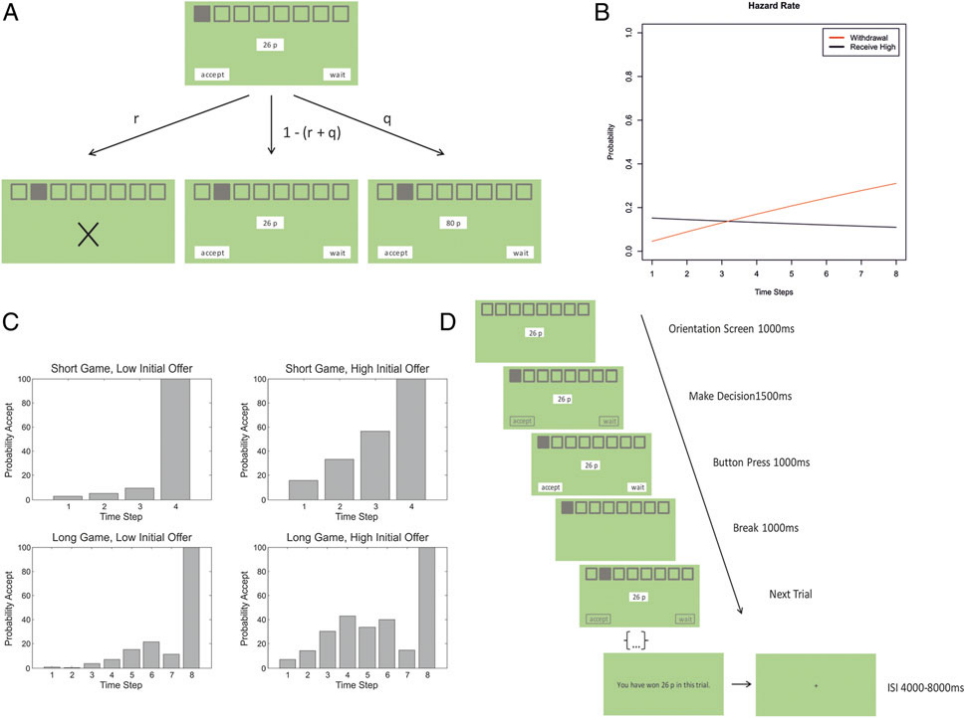
Experimental Design. (*A*) Each game comprised a specified number of trials (discrete time steps). On each trial, subjects had to decide whether to accept a current offer or wait for the next trial. If subjects decided to wait, the low offer could be withdrawn with probability *r*, it could be replaced by a high offer with probability *q* or could be carried over to the next trial with probability 1 − (*r* + *q*). (*B*) The withdrawal probability increased, whereas a high offer became less likely with each successive trial. (*C*) Average probabilities of accepting the initial offer at each trial (note that *P*_wait_ = 1 − *P*_accept_)., indicating that subjects were prepared to wait for the high offer (except at the last time step). The decrease in acceptance-probability at the seventh trial in long games is not due to an actual decreased propensity to accept but due to very few data points for these trials. (*D*) Structure of a game in the scanner: Each game started with an orientation screen, specifying the number of trials and the amount of the initial offer. At each trial, subjects had a brief period to make a decision, followed by a response period. Following a short break, the next offer was presented. After the last trial, the break-screen was replaced by a screen indicating the win amount on this game, followed by a jittered interstimulus interval (ISI)—before the next game.

To summarize, our limited offer task corresponds to a sequential choice paradigm, were subjects had to decide whether to accept an initial offer or wait for a higher offer (Fig. 1*A*). Each game comprised a specified number of trials, with a trial-specific probability of offer withdrawal and high offer. These probabilities were defined as hazard rates, such that withdrawal became more likely over time and the high offer became less likely (Fig. 1*B*). At the end of each game, participants were shown the outcome (Fig. 1*D*) and the total winnings determined the payment they received at the end of the experiment. Crucially, in this task, precision reflects trial-by-trial changes in the confidence about receiving the high offer (as discussed below), which makes this task ideally suited to investigate the neuronal mechanisms underlying the dynamics of precision.

### Scanning

In the scanner, each game started with an orientation screen (for 1000 ms) specifying the amount of the initial offer and the number of trials (i.e., the length) for this particular game. To ensure consistent responses and to differentiate motor responses from decision processes, subjects were given 1500 ms to think about their choice (without being able to respond), followed by a response period of 1000 ms, during which they made a button press. A 1000-ms interval—in form of a blank screen—was inserted before the next trial. When subjects failed to choose in the given time, the offer was withdrawn. Each game was followed by an interstimulus interval that was jittered between 4000 and 8000 ms. We counterbalanced the sides on which the options “wait” and “accept” would appear on the screen.

### Imaging Data Acquisition and Preprocessing

Scanning took place at the Wellcome Trust Centre for Neuroimaging, acquiring *T*_2_*-weighted echo planar images (EPIs) using a 3-T Trio Siemens scanner and a 32-channel head coil. We acquired a partial volume consisting of 42 3-mm slices in descending order (echo time: 0.065 ms, repetition time: 2.940 ms) at an angle of 30° in the anterior–posterior axis to optimize sensitivity to OFC (Deichmann et al. 2003). Images were acquired with a field of view of 192 × 192 mm (matrix 64 × 64) resulting in a notional in-plane resolution of 3 × 3 × 3 mm. In each session, 364 volumes were acquired and all subjects completed 3 sessions except subject 5, who only completed 2 sessions due to equipment failure. Five whole-brain EPIs with identical scan parameters were collected before starting the task in order to optimize co-registration. Foam head-restraint pads were used to minimize head movement and an MR-compatible button box recorded right index and middle finger presses to select “wait” or “accept.” Respiratory and cardiac activity were measured and used as covariates in the imaging analysis—to increase the signal-to-noise ratio. Whole-brain multiparameter maps were collected for anatomical localization using a 1-mm isotropic resolution (Helms et al. 2009)—allowing for precise localization of midbrain areas.

Preprocessing and statistical analysis was performed using SPM12b (Wellcome Trust Centre for Neuroimaging, London, UK, http://www.fil.ion.ucl.ac.uk/spm). The first 6 images were discarded to accommodate *T*_1_ relaxation effects. Functional images were unwarped using fieldmaps generated by the Fieldmap toolbox as implemented in SPM12b (Hutton et al. 2002). EPIs were then realigned to the mean image of each session and co-registered with the MT-weighted structural image. The DARTEL toolbox (Ashburner 2007) was used to normalize the EPIs and co-registered structural scans to MNI space—allowing the comparison of data between subjects in a common anatomical space.

### Computational Modeling

We assume that agents are equipped with a generative model *m* relating observations *O_t_* to (hidden) states *S_t_* and control states (i.e., sequences of actions and their consequences) *U_t_* at each trial t∈T, where a sequence of control states is called a policy π:

(1) $$P(\tilde{o},\tilde{s},\pi ,\gamma |\tilde{a},m)=P(\tilde{o}|\tilde{s})P(\tilde{s},\pi |\gamma ,\tilde{a})P(\gamma |m)$$

In this generative model, the first term of the right side of the equation represents the likelihood that informs state estimates, the second represents empirical priors over states and policies and the last term represents a prior over precision *γ*. The ∼ notation denotes sequences over time. Note that, in this task, there was no uncertainty about the mapping between observations and (hidden) states.

Inference about policies provides the agent's belief that a particular policy will be enacted, which can be expressed as:

(2) $$\text{ln}\;P(\pi |s_{t})=\gamma \cdot Q(\pi |s_{t})$$

This means that the agent's belief that a particular policy will be enacted depends on a precision (*γ*) weighted value (*Q*) of this policy, given the state at the current trial (*S_t_*). Actions or choices are sampled from the posterior marginal over the current control state.

Intuitively, a policy is valuable if it leads to outcomes that are close to desired outcomes. Formally, this means that a valuable policy minimizes the KL divergence (relative entropy) between a probability distribution over outcomes under the policy in question and a distribution over states that the agent believes it will (desires to) occupy:

(3) $$Q(\pi |s_{t})=-D_{\mathrm{KL}}[P(s_{T}|s_{t},\pi )||P(s_{T}|m)]$$

This divergence can be understood as the mismatch between states the agent believes it should end up in and states it is likely to end up in. The first probability distribution (in the KL divergence) represents the likelihood of final outcomes given the current state and policy, and therefore represents an empirical prior about the likely outcomes of a game, conditioned on a particular state and policy. The second distribution does not depend on any state or policy but represents the agent's prior belief about which state it will end up in, conditioned only on its model of the environment (goals). These goals map real world commodities (in our case: monetary offers) to an internal representation of utilities or prior beliefs about occupying different states (that are associated with different commodities):

(4) $$u(s_{T}|m)=\ln\;P(s_{T}|m)=\ln(\sigma ([\text{commoditi}{\text{y}}_{s}\cdot \kappa ]))$$

where the *κ* parameter represents the agent's sensitivity to the differences in the utilities of the different outcome states and *σ* is the softmax function, ensuring that the prior probabilities add to one.

Crucially, the value of a policy can be re-written as

(5) $$Q(\pi |s_{t})=H[P(s_{T}|s_{t},\pi )]+\underset{s_{T}}{\sum }P(s_{T}|s_{t},\pi )\cdot u(s_{T}|m)$$

This equation shows that the value of a policy can be decomposed into entropy and expected utility terms. The entropy term increases if the agent visits several different or novel states (i.e., increases its entropy over states), whereas the expected utility term connects to classical theories from behavioral economics—and is maximized if the final state with the highest expected utility is attained. In other words, a valuable policy maximizes the entropy over the final states and the expected utility over those states. This decomposition of the value of a policy fits neatly with accounts of intrinsic and extrinsic reward (Luciw et al. 2013) and connects to classic notions of exploration and exploitation (Cohen et al. 2007; Daw 2009). Here, increasing the entropy over goal states corresponds to the concept of a novelty bonus (Kakade and Dayan 2002) or information gain, whereas maximizing expected utility corresponds to exploitation. In what follows, we will contrast this (KL optimal) formulation with classical schemes that only consider the expected utility. In the current setting, expected utility contributes to prior beliefs about behavior that are updated to form posterior beliefs on the basis of observed outcomes. Later, we will use the posterior distribution over actions as a measure of choice uncertainty or conflict.

Equation (2) shows that the probabilistic selection of a policy depends not just upon its relative value but also precision. In other words, to minimize free energy, action selection needs to be modulated by contextual information, which is accommodated by precision. Prior beliefs over policies take the form of a probability distribution with *γ* or precision acting as an (inverse) temperature parameter—therefore modulating the sensitivity of an agent to differences in the value of different policies. It can be thought of as representing the confidence in the most valuable policy, that is, the confidence that one will indeed attain preferred goals, which has to be updated on the basis of observations.

In our scheme, approximate inference is performed using Variational Bayes (Attias 2000; Beal 2003), which leads to precision updates with the following form:

(6) $$\hat{\gamma }=\frac{\alpha }{1-{\hat{\pi }}^{T}\cdot Q\cdot {\hat{s}}_{t}}.$$

Posterior precision follows a standard gamma distribution: P(γ|m)=Γ(α,β=1) with scale parameter α. The expected precision is updated at each trial depending on the inferred policy, choice values, and expected current (hidden) state. This equation implies that precision falls when the initial offer is withdrawn, because the confidence in receiving the high offer is minimal, and increases when the high offer is accepted—because the confidence in reaching the goal (of ending with the highest offer) becomes maximal. Equation (6) has an important interpretation; it says that (changes in inverse) precision equals (changes in negative) expected value, where expected value is the value expected under the current state and policy ${\hat{\pi }}^{T}\cdot Q\cdot {\hat{s}}_{t}$. From the point of view of the precision hypothesis, this may explain why dopamine firing has been interpreted in terms of RPE. See Friston et al. (2013) for a detailed description of the computational scheme and its application to the paradigm used in this paper.

### Behavioral Analysis and Modeling

Given the form of a generative model, one can use observed choice behavior to estimate the parameters of the model that best accounts for the choices of each subject. These parameters could include the hazard rate or prior beliefs about precision used by a particular individual. We estimated 3 parameters, based upon subject's performance, using the Hierarchical Gaussian Filtering Toolbox to compute maximum a posteriori estimates (Mathys et al. 2011; Mathys 2012). Maximum a posteriori estimation maximizes the evidence for posterior estimates of parameters given observed responses and prior distributions over unknown parameters, generally specified as lognormal priors. For each subject, we estimated the *α* or prior precision, a hazard rate, and *κ*, the sensitivity to commodity values. The prior means for α were 8 (with a standard deviation of 1/16), 1/22 for the hazard rate (with the estimate bounded between 0 and 1 by a logit function over the Gaussian prior) and 1 for *κ* (with a standard deviation of 1/16). The likelihood of the particular choice, for a given set of parameters, was obtained using the spm_MDP_offer.m routine as implemented in the DEM toolbox of SPM12b (Wellcome Trust Centre for Neuroimaging, London, UK, http://www.fil.ion.ucl.ac.uk/spm). This routine uses variational Bayes to provide distributions over policies that minimize free energy using the Markov decision process implicit in the probability transitions describing our task (see above). Only trials in which subjects decided to wait or accept the initial offer were used for behavioral model comparison and parameter estimation, as only these trials report motivated choices.

### Imaging Data Analysis

A standard (event-related) statistical parametric mapping (SPM) analysis was used to test for specific task-effects and belief updates, where each trial was treated as a separate event. Our regressors modeled onsets of the initial screen, outcome screen and all trials of a game—as well as the onsets of trials in which subjects made a motor response. We included 5 parametric regressors for precision, the updates (or first derivative) of precision, choice conflict, the updates (or first derivative) of choice conflict, and the length of a game (long and short). Values for precision and choice conflict were set to zero whenever a final state (having accepted an offer or it being withdrawn) was reached, assuming no further decision related processing. Choice conflict was defined as the entropy of the posterior distribution over actions, which increases when the probabilities for wait and accept (as estimated by the model) are similar (thereby reflecting an internal conflict or ambiguity about the decision) and decreases when the probabilities are very different (i.e., when we assume little cognitive conflict concerning the 2 choices).

We included temporal derivatives (first-order differences) as covariates of no interest to account for slice timing effects and applied a high pass filter (using a 128-s cutoff) to remove low-frequency drifts. Finally, we accounted for physiological noise with respiratory, cardiac, and motion regressors. An AR(1) model was used to model serial correlations in the fMRI time series.

We used a standard summary statistic approach to random effects analysis: Second (between-subject)-level responses were tested by performing one-sample *t*-tests on the linear contrasts of the estimated responses from the first (within-subject)-level general linear model analyses. We corrected for multiple comparisons in the ensuing SPM using random field theory (for a whole-brain search and region-of-interest [ROI] analyses).

We performed small-volume-corrected ROI analyses of midbrain responses in the SN/VTA. The ROI for SN/VTA was drawn manually on our sample mean structural image, which can be distinguished from surrounding areas as bright stripe (Düzel et al. 2009). The ROIs for specific effects of choice conflict were obtained from the WFU pickatlas (Maldjian et al. 2003) and the AAL atlas (Tzourio-Mazoyer et al. 2002). All (corrected) peak-level results reported survived a height threshold of *P* = 0.001 (uncorrected).

## Results

### Choice Behavior

Participants were sensitive to differences in experimental factors (number of trials and magnitude of the initial offer) and adjusted their choice behavior accordingly. When comparing short (4 trials) and long (8 trials) games with low (between 9 and 15 pence) and high (between 29 and 35 pence) initial offers, we found that high initial offers led to an earlier acceptance, compared with low initial offers, a distinction more pronounced in long compared with short games (Fig. 1*C*). This was confirmed by fitting a general linear model of acceptance latency with the predictors’ game length, initial offer, and their interaction. This showed that acceptance latency increased in longer games (*b* = 0.57, *F*_1,995_ = 149.34, *P* < 0.01), decreased for high compared with low initial offers (*b* = −0.83, *F*_1,995_ = 311.94, *P* < 0.01), with a larger difference between high and low initial offers in long compared with short games (*b* = −0.26, *F*_1,995_ = 31.04, *P* < 0.01). The mean acceptance latency for short games was 3.52 trials (SD = 1.01) for low and 2.39 (SD = 1.06) for high initial offers and for long games 5.19 (SD = 1.92) for low and 3.01 (SD = 1.55) high initial offers, respectively.

### Modeling Subject-Specific Behavior

**Figure 2.**
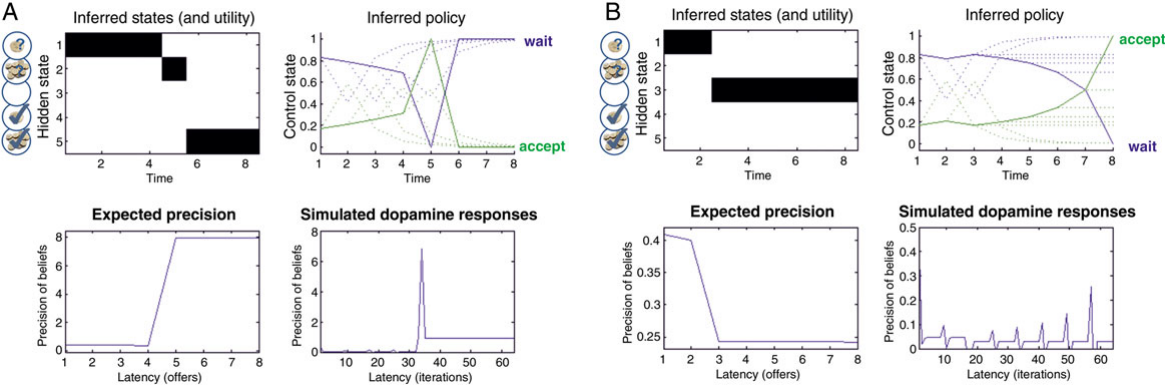
Simulation of state-dependent changes of expected precision and putative dopaminergic responses. (*A*) Receiving the high offer (left upper panel) produces a sharp increase in precision (lower left panel) and simulated dopaminergic firing (lower right panel). The high offer induces a change in policy and compels the agent to accept (“switch,” upper right panel). (*B*) Same format as in (*A*), but illustrating the withdrawal of an initial offer, leading to a decrease in precision and a consequent dip in dopaminergic firing.

Our (Bayes optimal) scheme deploys a standard gamma prior over precision with scale parameter α. Precision is then updated at each trial according to the current state, with a sharp increase after a high offer and a decrease when an offer is withdrawn (because the confidence in reaching the desired goal is maximal and minimal, respectively). The behavior of expected (posterior) precision for these 2 outcomes is illustrated in Figure 2.

**Figure 3.**
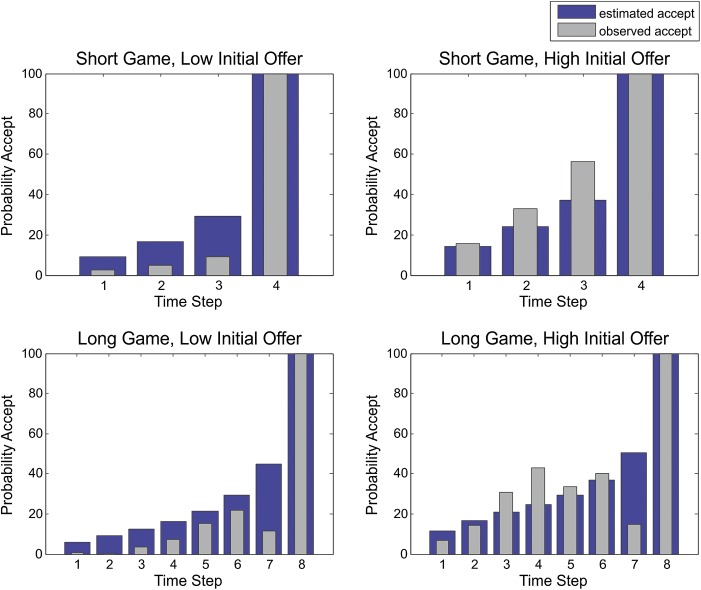
Observed and estimated acceptance probabilities as predicted by our model (note that *P*_wait_ = 1 − *P*_accept_). These estimates are based upon maximum a posteriori values for prior precision, the hazard rate, and sensitivity to differences in monetary cues. Note that the observed acceptance probabilities are identical to the ones shown in Figure 1*C*.

Using the observed probability of accepting over successive trials for each subject, we estimated: (*α*), a hazard rate (*r*), and a monetary sensitivity parameter (*κ*) for each subject individually. These parameters encode the subject's prior expectations about precision, hazard rates, and their sensitivity to monetary cues, respectively. The parameters from this behavioral modeling are shown in Table 1. Their intercorrelations suggested a substantial and unique contribution of each parameter to behavior (*r*(*α*, *κ*) = 0.48, *r*(*α*, hazard rate) = 0.61), *r*(*κ*, hazard rate) = 0.66). The overall variance in observed choice behavior explained by the behavioral modeling was *R^2^* = 0.81 and predicted choice probabilities closely resembled observed choice probabilities (Fig. 3). Although the empirical results suggest a slight dip in the probability of accepting in penultimate trials, this merely reflects the fact that there were very few trials with these long deferments.

**Table 1:** Individual model parameters and their associated accuracy expressed as the proportion of explained variance

Subject	*α*	*κ*	Hazard rate	*R*^2^
1	8.0044	1.0000	0.4576	0.4300
2	18.4709	1.0004	0.4358	0.6237
3	11.0772	1.0010	0.4260	0.4623
4	8.7165	0.5916	0.5291	0.1417
5	22.3780	1.0001	0.3608	0.8716
6	7.7290	1.0000	0.5095	0.3145
7	23.1915	1.0000	0.3585	0.5262
8	12.0402	1.0001	0.3851	0.2372
9	31.9133	1.0001	0.2554	0.9840
10	20.5454	1.0001	0.3842	0.6727
11	21.1646	1.0002	0.4219	0.7541
12	20.0900	0.9999	0.4013	0.6727
13	25.0789	1.0001	0.2885	0.6613
14	6.6711	1.0016	0.5537	0.2332
15	23.8371	0.9998	0.3283	0.7259
16	24.8066	1.0001	0.2921	0.8018
17	10.2983	0.9958	0.4321	0.2936
18	34.1881	0.6106	0.3401	0.9011
19	13.6716	0.9998	0.4675	0.5423
20	14.2230	0.9999	0.4033	0.5423
21	30.8221	1.0000	0.2750	0.9410
22	11.1589	0.9978	0.4665	0.7390
23	33.8191	0.5764	0.5752	0.6675
24	11.2746	1.0000	0.4888	0.4742
Average	18.55	0.95	0.41	0.8139

Note that our modeling maximized model evidence (by minimizing variational free energy) which accounts for model complexity in addition to accuracy.

Even though the model fits were impressive for such a simple model, there may be room for improvement. This speaks to further model comparisons in the future—using more complicated priors or using more free parameters; for example, including a nonspecific prior cost for waiting or optimizing the subjects' estimates of transition probabilities. However, with an *R*^2^ of over 80%, we considered the current model sufficient for our purposes.

The estimated parameters characterize subject-specific traits that model between-subject variability in choice behavior. Of particular interest here is the trait or prior expectation about precision, namely α. The corresponding state or “posterior” expectations about precision reflect trial-by-trial changes in confidence about waiting for a high offer, that are nuanced by these “prior” beliefs. If prior precision is high, subjects tend to wait longer for a high offer. Accordingly, the estimate of each subject's prior precision correlated positively with the average number of trials waiting for a high offer (*r* = 0.45, *P* = 0.026), and negatively with the total number of games in which the initial offer was accepted (*r* = − 0.45, *P* = 0.026).

### Model Comparison

In our theoretical formulation, policy selection is based upon minimizing the KL divergence between the distribution over states the agent wants to be in, and those it believes it can reach from the current state. This divergence can be decomposed into the entropy of the probability over final outcomes and an expected utility term. This is important because it suggests that choice behavior will maximize both the entropy over outcomes and their expected utility (Schwartenbeck et al. 2013). This contrasts with classical utilitarian approaches, which assume that choices maximize expected utility alone. To disambiguate between these accounts of choice behavior, we used the behavioral data to compare active inference, in which subjects maximize both entropy and expected utility, with a model wherein subjects maximized expected utility alone. In other words, we compared models in which the KL divergence was replaced by expected utility. Random effects Bayesian model comparison (Stephan et al. 2009) revealed strong evidence in favor of the active inference model (exceedance probability *φ* > 0.99). Ignoring the complexity of the models and just focusing on the accuracy or explained variance as represented by *R*^2^ values, we found that the active inference model explained more variance in 21 of 24 subjects, even though the overall difference in accuracy between the 2 models was small ($R_{\mathrm{active}\;\mathrm{inference}}^{2}=0.81,\;R_{\mathrm{expected}\;\mathrm{utility}}^{2}=0.78$). The increase in accuracy was statistically significant in a two-tailed Wilcoxon signed rank test (*z* = − 3.8857, *P* = 0.0001).

Clearly, the agreement between the behavioral results and theoretical predictions depends in part on the hundred percent probability of accepting on the last trial. To ensure that this characteristic (of both KL optimal and classical utilitarian schemes) was not confounding the model comparison, we repeated the analysis without forcing the model to accept at the last time step. In this analysis, the model fit was *R*^2^ = 0.75 and the exceedance probability for the KL scheme over the classical scheme was again *φ* > 0.99.

Accordingly, we used the active inference model to predict trial-by-trial variations in neuronal responses.

### Model-Based Correlates in the Brain

To generate suitable explanatory variables (stimulus functions) to model fMRI responses, we used trial-by-trial variations in posterior expectations, based upon the subject-specific priors above. This approach highlights the mediating role of computational modeling, in linking the behavioral responses of each subject to their physiological responses—as encoded by a conventional general linear convolution model of fMRI time series. Here, we focused on choice conflict and confidence, measured as the entropy over alternative choices (wait vs. accept) and expected precision, respectively.

**Figure 4.**
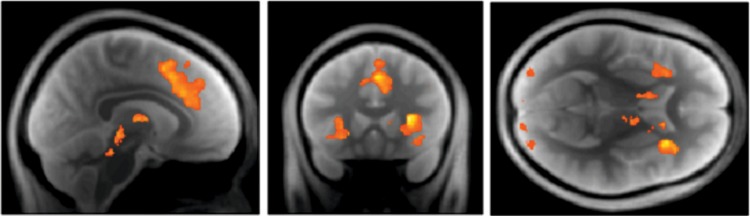
Effects of choice conflict defined as the entropy of the probability distribution over the 2 alternative actions (wait vs. accept). SPM thresholded at *P* < 0.005 (uncorrected) and masked for the midbrain for display purposes.

Testing for neuronal correlates of conflict identified the left and right insular (left: peak MNI coordinates: −28 24 3, cluster size = 2158, *P*_peak_ < 0.003, *T*_peak voxel_ = 8.60; right: peak MNI coordinates: 30 21 9, cluster size = 3029, *P*_peak_ < 0.001, *T*_peak voxel_ = 10.04) and anterior cingulate cortex (ACC) (peak MNI coordinates: 5 18 45, cluster size = 4372, *P*_peak_ = 0.002, *T*_peak voxel_ = 8.62, Fig. 4). These significant (whole-brain-corrected) results imply that activity in these regions increased with choice conflict and neatly connects to previous results on the neuronal mechanisms underlying conflict and uncertainty (Brown and Braver 2007, 2008; Platt and Huettel 2008). Choice conflict—as estimated by our model—therefore predicts neuronal activation in regions commonly activated by paradigms involving conflict and is in line with theories associating the ACC with conflict monitoring (Botvinick et al. 2001; Botvinick et al. 2004). We did not identify any effects of game length on trial-specific responses.

### Precision Updates and the Dopaminergic Midbrain

**Figure 5.**
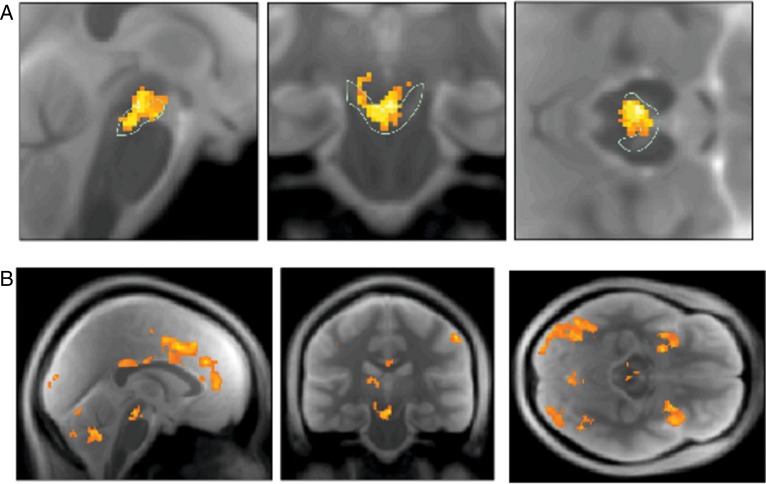
Neuronal correlates of expected precision in the brain. (*A*) Using a hand-drawn ROI for the SN/VTA, we detected substantial effects for expected precision in both the substantia nigra as well as the ventral tegmental area: SPM thresholded at *P* < 0.005 (uncorrected) and masked for the midbrain for display purposes. SPM thresholded at *P* < 0.005 (uncorrected) and masked for the midbrain for display purposes. (*B*) At the whole-brain level, we found distributed and strong effects of precision in prefrontal, inferotemporal, and parietal regions as well as in the striatum and anterior insular cortex. SPM thresholded at *P* < 0.005 (uncorrected) and masked for the midbrain for display purposes.

Our main question concerned whether expected precision predicted activity in dopamine-rich brain regions. To answer this, we used a (small-volume-corrected) analyses based on an anatomical ROI isolating the SN and VTA. The SN/VTA area contains ∼90% of all dopaminergic projection neurons in humans and nonhuman primates (Düzel et al. 2009). Our restricted small-volume analysis using the SN/VTA ROI strongly supported the hypothesis that dopaminergic midbrain activity encodes expected precision in the SN (peak MNI −10 −22 −11, *P*_peak_ = 0.008, *T*_peak voxel_ = 4.58) and the VTA (peak MNI 5 −19 −15, *P*_peak_ = 0.005, *T*_peak voxel_ = 4.79; peak MNI −3 −21 −15, *P*_peak_ = 0.006, *T*_peak voxel_ = 4.78) (Fig. 5*A*). Equivalent tests for the effects of changes in expected precision did not survive whole-brain correction.

Finally, to test the hypothesis that precision provides a sufficient account of midbrain responses, we repeated the above analyses but including expected value (under the utilitarian model) and the current monetary offer (reward) of the current trial as additional explanatory variables. Tests for the effects of expected value or reward did not survive correction for multiple comparisons in the midbrain ROI. While these results do not imply that expected value or reward do not contribute to midbrain responses, we can say that precision provides a sufficient account of any of observed midbrain responses that could have been explained by expected value or reward. Interestingly, however, despite the colinearity between precision and expected value (and current reward), the effect of precision was still significant in the VTA (peak MNI 5 −21 −17, *P*_peak_ = 0.035, *T*_peak voxel_ = 3.89; peak MNI −4 −25 −17, *P*_peak_ = 0.039, *T*_peak voxel_ = 3.84; corrected for the midbrain ROI) and showed trend significance in the SN (peak MNI −10 −25 −12, *P*_peak_ = 0.070, *T*_peak voxel_ = 3.53; corrected for midbrain ROI). This suggests that not only is precision sufficient to explain midbrain responses, it explains components that cannot be explained by expected value (or current reward) alone.

### Effects of Precision on Other Brain Regions

Although our primary hypothesis concerned precision-related responses in dopaminergic midbrain regions, the neurobiological implementation of active inference using variational Bayes also makes strong predictions about precision-related effects in other brain systems (see Friston et al. (2013)). Crucially, it predicts that expected precision should also modulate Bayesian belief updates in systems inferring hidden states of the world—including states that entail control or action. Physiologically, this means that we would expect to see augmented trial-related responses not just in the source of dopaminergic projections but also in their targets—targets that are involved in processing visual information and simulating the outcomes of alternative policies (planning).

We detected significant precision-related responses in several regions, after correction for a whole-brain search, that included visual and inferotemporal cortical regions engaged during perceptual categorization, and prefrontal regions associated with delay period activity and planning (see Table 2 and Fig. 5*B*). Interestingly, these prefrontal regions extended to anterior insular cortex consistent with an affective (autonomic) component to neuronal inference. Although we do not discuss the functional anatomy of these results in any detail we note that precision-related effects are distributed and are consistent with the neuromodulation of belief updates in brain systems involved in perceptual inference (Rao and Ballard 1999), action selection (Cisek and Kalaska 2010), and (more speculatively) interoceptive inference (Seth et al. 2011). Of the regions implicated in our analysis, the anterior insular cortex, frontal regions such as the anterior cingulate cortex and the ventral and dorsal striatum have established dopaminergic projections.

**Table 2:** Effects of precision on the whole-brain level (reported on the cluster level for *P* < 0.001 uncorrected)

Region	Cluster size	FWE-corrected *P*-value	*T*-value of peak voxel	Peak coordinates (MNI)		
				*x*	*y*	*z*
Cerebellum	243	0.011	4.73	−9	−82	−35
	306	<0.001	5.83	0	−55	−36
Sensory (visual)	3390	<0.001	7.36	36	−93	−6
	5260	<0.001	6.41	−25	−97	−12
Parietal	2928	<0.001	6.89	−28	−54	47
	306	0.003	4.94	−6	−69	38
Frontal	2463	<0.001	6.97	44	12	44
	384	<0.001	6.93	−51	6	36
Cingulate	4002	<0.001	6.13	−3	5	32
Midbrain/striatum/insular cortex	2560	<0.001	6.40	−31	−3	−5
	1752	<0.001	7.07	23	−3	11

## Discussion

Dopamine activity in humans and other animals is linked to a variety of apparently diverse functions (Düzel et al. 2009). This speaks to the need for theories that assign dopamine a more general role—as opposed to relating it exclusively to reward (Pessiglione 2006; Schultz 1998), salience (Berridge 2007, 2012), novelty (Bunzeck and Düzel 2006; Redgrave and Gurney 2006), working memory (Williams and Goldman-Rakic 1995), or learning (Steinberg et al. 2013). We have proposed that it plays a generic role in modulating or selecting the neuronal messages that are exchanged during active Bayesian inference, most concisely formalized as encoding precision in predictive coding (Friston 2005; Friston et al. 2012). This formulation of dopamine arises naturally from treating the brain as hierarchical Bayesian inference machine that minimizes surprise or free energy. Here, we show that the responses of a dopamine-rich region of the brain are predicted by this generic but computationally specific role in the context of decision making.

In the setting of choice behavior, this scheme implies that dopamine reports the expected certainty or precision of beliefs about policies and thereby the confidence a policy will deliver an outcome during purposeful and planned behavior. This confidence must adapt with context so as to minimize free energy. The optimization of precision has both trait and state aspects namely, prior (trait) and posterior (state-dependent) precision. Individuals with a higher trait-precision wait longer for desired outcomes (e.g., the high offer) and accept initial offers less frequently. They are therefore less stochastic in their behavior, showing more patience when waiting for the high offer. Posterior or context-sensitive precision reflects the confidence in reaching a desired goal, conditioned on the current state. This means that it resembles a reward prediction signal as posited by reinforcement learning: see below and (Schultz et al. 1997; Fiorillo 2003). In the context of reinforcement learning paradigms, the RPE now becomes the change in confidence about receiving anticipated rewards. In a nonreward context, expected precision reflects the novelty associated with increased entropy over potential outcomes. This is important because dopamine discharges have also been associated with salience and novelty (Bunzeck and Düzel 2006; Redgrave and Gurney 2006; Berridge 2007, 2012).

Our results provide behavioral and neuronal validation of an active inference formulation of optimal behavior. Bayesian model selection suggests that this scheme offers a better (and more accurate) account of behavior than a model based solely on expected utility. Explaining behavior in terms of minimizing the KL divergence, rather than simply maximizing expected utility also provides a principled explanation for the tradeoff between exploration and exploitation (Schwartenbeck 2013) and can be extended to explain phenomena such as Herrnstein's matching law (Herrnstein 1961). The entropy term is a simple and necessary component of KL control (Ortega and Braun 2013) that can be thought of as “keeping one's options open.” This component may also provide a formal basis for heuristics like the exploration bonus (Kakade and Dayan 2002).

Clearly, there are many ways to model our paradigm. However, the active inference framework comfortably accommodates many standard models as special cases. In particular, it contextualizes and provides a possible biological implementation for models based on maximizing expected value, such as model-based or hierarchical reinforcement learning (Ribas-Fernandes et al. 2011; Huys et al. 2012). Active inference absorbs rewards into prior beliefs by placing choice behavior in the broader context of minimizing surprise (i.e., maximizing model evidence or marginal likelihood). In our scheme, agents seek out states they believe they should occupy, hence desirable (rewarding) states are unsurprising states and undesirable (unrewarding) are surprising. It is easy to see that, by minimizing surprise, agents will seek out familiar and rewarding goals. Casting decision making as active Bayesian inference may help to contextualize the interpretation of utilitarian models. For example, through the monotonic relationship between expected value and precision: see Equation (6) and Friston et al. (2013). Furthermore, our framework provides straightforward applications to tasks that do not invoke explicit rewards or support value learning, such as the urn task (FitzGerald et al. in review).

In the introduction section, we distinguished between inference and learning—framing the current game as an inference problem. Our primary motivation was to manage expectations about comparisons with classical schemes based upon RPE. The question, “how does this scheme compare with RPE formulations?,” raises some fundamental issues about the nature of formal models and normative schemes that we now briefly consider.

It is useful to emphasize the distinction between inference and learning problems (Botvinick and Toussaint 2012). Pure inference (or planning) problems require an agent to find an optimal solution (behavior) based on a set of observations and a generative model of the environment. Learning problems, in contrast, require the agent to update the parameters of its generative model—or indeed learn the model structure through model comparison. In the context of planning, the simplest way to optimize behavior is to evaluate the outcomes of allowable policies and select the policy that maximizes expected value. This is effectively what the variational Bayesian scheme we have used does. This scheme makes it clear that planning requires the representation of future states—and allows one to cast optimal control problems as pure inference problems (Botvinick and Toussaint 2012; Friston 2011). The alternative would be to formulate the planning problem as a control problem and solve the associated dynamic programming or Bellman equation using backwards induction. In other words, assume that the policy is specified by a value function and compute the value function by working backwards from the final (valuable) state. In principle, variational Bayes and backwards induction should give similar answers; however, there is no neuronally plausible message-passing scheme for backwards induction.

Note that variational Bayes and backwards induction do not call on RPE, which arises in reinforcement learning schemes where the value of actions and states are learnt through repeated experience (Sutton and Barto, 1998). In brief, reinforcement learning schemes such as Rescorla-Wagner, Q-learning, and SARSA try to solve the appropriate Bellman optimality equations using variants of the Robbins Munro algorithm (Robbins and Munro 1951). In other words, they try to estimate a value function of states (or state-action pairs) using stochastic approximation. In these schemes, a running estimate is updated using the RPE multiplied by a small number (the learning rate). Under certain (ergodic) assumptions, this scheme is guaranteed to converge to the true value function, when on average, the prediction error is zero. However, RPE cannot be used in the context of the (partially observed) Markov decision problems considered above. This is because optimal choices have to be inferred for each game—precluding stochastic approximation over multiple games.

When one considers these issues in operational terms, it becomes clear that RPE is an auxiliary variable associated with stochastic approximation schemes for learning value functions. Given this insight, what would be an alternative to variational message passing? This is a difficult question to answer, because the natural response would be some form of optimal control; for example, solving the Bellman equation using backwards induction. However, this is formally equivalent to the solution of the equivalent inference problem using Bayesian filtering (or smoothing)—either exactly or approximately (Todorov 2008; Friston 2011; Tassa et al. 2011).

In summary, active inference offers a process theory, describing the real-time neuronal message passing that underlies optimal choice behavior. This formulation equips the normative account (i.e., minimizing variational free energy or maximizing expected value) with a biologically plausible implementation—an implementation that makes some clear predictions about measurable behavioral and neuronal responses.

In short, unless one thinks the brain uses a stochastic approximation scheme to make optimal decisions on the basis of a single trial or sequence, there is no reason to invoke RPE. Indeed, all the physiological evidence in support of RPE responses during learning was used to motivate an understanding of dopamine as encoding precision during inference (Friston et al. 2013). Perhaps, the deeper question is not how the current scheme compares with classical (reinforcement learning) schemes; but rather what are the (biologically plausible) alternatives one could consider?

Furthermore, the notion that dopamine reports precision or confidence in the context of planning or inference fits comfortably with several established ideas about the role of dopamine during motivated behavior. Important examples here include the notions of “wanting” (e.g., Berridge 2007, 2012) and vigor and effort (e.g., Salamone and Correa 2012). Incentive salience is particularly interesting because of the close relationship between salience and precision—especially in the domain of visual attention. It may not be straightforward to cast wanting or incentive salience in terms of formal (active) inference; however, the close relationship between the salience of incentivizing cues and the confidence or precision in beliefs that cued outcomes can—or will be—attained is self-evident.

Minimizing surprise about future outcomes generally implies the minimization of a relative entropy, or KL divergence, between likely and desired outcomes. Active inference connects to established accounts of KL control but places KL divergence in the setting of inference. This eliminates the need for ad hoc parameters in that the softmax temperature parameter of classical (utilitarian) approaches is replaced by a precision that is itself optimized. Our fMRI results suggest this optimization may provide a simple account of dopaminergic midbrain responses.

We have provided evidence that precision may be encoded by activity in midbrain regions that are associated with dopaminergic activity (Düzel et al. 2009; Guitart-Masip et al. 2011). However, in biologically plausible implementations of active inference, variational updating of precision and inferred (control) states are widely distributed. For example, Bayesian updates about hidden states of the world may be encoded by prefrontal neuronal activity but this in turn depends on perceptual categorization in lower visual and inferotemporal regions. All of these regions have to inform precision updates in the dopaminergic midbrain, which then optimize perceptual inference and policy selection. This is consistent with our findings of prefrontal, striatal, and insular responses being modulated by expected precision—and emphasizes the distributed causes and consequences of (putative) dopaminergic activity.

Establishing a link between precision and dopaminergic activity provides insights into both the neurobiology of decision making as well as into its pathologies. The link between psychopathology and abnormalities of precision is a focus of much current research, and has been discussed in the context of a number of disorders including psychosis (Adams et al. 2013), Parkinson's disease (Frank 2005; Friston et al. 2012), functional disorders (Edwards et al. 2012), and autism (Pellicano and Burr 2012). Associating precision with dopaminergic firing also suggests the fruitful application of this sort of paradigm to conditions associated with pathologic decision making, such as obsessive–compulsive disorders or addiction. Intriguingly, both obsessive–compulsive disorders (Politis et al. 2013) and addiction (Diana 2011; Taber et al. 2012) have been associated with abnormal dopaminergic function. Indeed, this article is a prelude to an application of our paradigm to addiction—which we anticipate will be associated with abnormalities of dopaminergic and precision-related responses. In short, we will investigate specific characteristics of the generative model underlying addictive choice behavior to understand the behavioral and neuronal mechanisms that cause pathological decision making in addiction. We expect that addiction will be marked by a decreased prior precision, inducing more impulsive and habitual behavior. This also resonates with findings that addiction is associated with a diminished phasic response of dopaminergic neurons (Willuhn et al. 2014). Clearly, hyperdopaminergic states—associated with pathologically high levels of (prior) precision—may induce maladaptive conditions such as overly optimistic state inference (Friston et al. 2013) or failures of sensory attenuation in schizophrenia (Adams et al. 2013).

In conclusion, our findings associate dopaminergic rich midbrain activity with the expected precision of beliefs about controlled outcomes, namely the confidence of reaching a desired goal. The findings inform the ongoing discussion about the general role of dopamine in brain function as well as its relationship to a range of cognitive processes, such as working memory, reward prediction, salience, novelty, and learning as well as pathologies of decision making. The current study provides strong evidence that humans perform a rather sophisticated form of probabilistic reasoning, which critically involves the inference on the precision with which certain beliefs are held. Thus, our results are in line with the Bayesian brain hypothesis, casting the brain as a hierarchical Bayesian inference machine. Furthermore, our study provides behavioral and neuronal validation of active inference in decision making, and lays the groundwork for a unified understanding of how behavior is selected and contextualized by the brain through hierarchical Bayesian inference.

## Funding

This work was supported by the Wellcome Trust (Ray Dolan Senior Investigator Award 098362/Z/12/Z). The Wellcome Trust Centre for Neuroimaging is supported by core funding from the Wellcome Trust 091593/Z/10/Z. Christoph Mathys is supported by a Joint Initiative involving Max Planck Society and University College London on Computational Psychiatry and Aging Research. Funding to pay the Open Access publication charges for this article was provided by Wellcome Trust.
